# A fine-grained dataset for sewage outfalls objective detection in natural environments

**DOI:** 10.1038/s41597-024-03574-9

**Published:** 2024-07-02

**Authors:** Yuqing Tian, Ning Deng, Jie Xu, Zongguo Wen

**Affiliations:** 1 School of Environment, Tsinghua University, Beijing, 100084 PR China; 2 Changjiang Basin Ecology and Environment Monitoring and Scientific Research Center, Changjiang Basin Ecology and Environment Administration, Ministry of Ecology and Environment, Wuhan, 430010 China

**Keywords:** Environmental impact, Environmental impact

## Abstract

Pollution sources release contaminants into water bodies via sewage outfalls (SOs). Using high-resolution images to interpret SOs is laborious and expensive because it needs specific knowledge and must be done by hand. Integrating unmanned aerial vehicles (UAVs) and deep learning technology could assist in constructing an automated effluent SOs detection tool by gaining specialized knowledge. Achieving this objective requires high-quality image datasets for model training and testing. However, there is no satisfactory dataset of SOs. This study presents a high-quality dataset named the images for sewage outfalls objective detection (iSOOD). The 10481 images in iSOOD were captured using UAVs and handheld cameras by individuals from the river basin in China. This study has carefully annotated these images to ensure accuracy and consistency. The iSOOD has undergone technical validation utilizing the YOLOv10 series objective detection model. Our study could provide high-quality SOs datasets for enhancing deep-learning models with UAVs to achieve efficient and intelligent river basin management.

## Background & Summary

Much sewage and wastewater are being released into natural water bodies, resulting in water scarcity and environmental issues such as eutrophication, excessive metal contamination and plastic pollution^1–3^. Sewage outfalls (SOs), found extensively on both sides of the river, are specific channels for releasing pollutants from various sources of pollution into the water bodies^4^. Many managers recognize the importance of locating, obstructing, and regulating SOs to protect the natural water bodies^5,6^. High-resolution images are suitable for the analysis and interpretation of SOs. Currently, the interpretation must be performed by individuals with specialised environmental science knowledge. The over-reliance on professionals has several disadvantages, including the time-consuming and labour-intensive, which has impeded the widespread identification of SOs in large-scale river basins^7,8^.

Deep learning has advanced visual technology, enabling computers to acquire the expertise of image interpreters and become intelligent tools capable of detecting SOs objectives in large-scale river basins^9^. A high-resolution image set of SOs that can be used for advanced model training, validation, and testing is a necessary prerequisite for monitoring SOs to benefit from deep learning^10,11^. Therefore, a high-quality SOs image dataset is significant for engineers, scientists, and managers in SOs identification. However, there is no satisfactory dataset of SOs.

Several conducted research can serve as references for creating image datasets for SOs objective detection, such as those by Xu *et al*.^12^ and Huang *et al*.^10^. Xu *et al*. used the UAVs, which capture high-resolution images of SOs by operating at low altitudes. However, these photos are only about 600, which makes it challenging to meet the requirements of deep learning^12^. Huang *et al*. operated UAVs at a significant elevation and obtained around 7000 images of SOs^10^. Nevertheless, several disadvantages of these images lead to a diminished level of accuracy in identifying SOs, including (i) long-distance shooting leads to the size of the SOs in the field of view being too small to be identified; (ii) vertical photography makes it easy to ignore the sewage outlet with a small protruding amplitude. In recent years, China’s official administration has conducted a comprehensive survey of SOs to determine their exact spatial locations and capture images. This effort has accumulated a significant number of pictures. Nevertheless, these photos are unrefined and devoid of annotations, rendering them challenging to utilize directly for deep learning models. Fortunately, this study thoroughly examines these materials and establishes a standard SOs dataset. Moreover, the YOLOv10 series, one of the state-of-the-art target detection models, is used to evaluate the performance of the SOs dataset in this study^13^.

The main contribution of this study is the development of a high-quality dataset named the images for sewage outfalls objective detection (iSOOD) for the first time. The construction of iSOOD is determined by the following criteria^14^:

(i) **Diversity**. Images are acquired in diverse geographical locations and lighting situations, encompassing various kinds of SOs;
(ii) **Accuracy**. Our research team has completed and repeatedly checked the annotation work to ensure accuracy.
(iii) **Consistency**. The annotation of the images follows the standard YOLOv10 format^15^;
(iv) **Extensibility**. The images match specific attribute information, such as the category.

The iSOOD dataset consists of 10481 images and 10481 records of specific attribute information. The purpose is to encourage researchers to create advanced deep learning models using this iSOOD dataset and collaborate with us. Our mission is to promote the implementation of advanced detection technologies for SOs globally to enhance the intelligent management capabilities of river basins.

## Methods

**Fig. 1:**
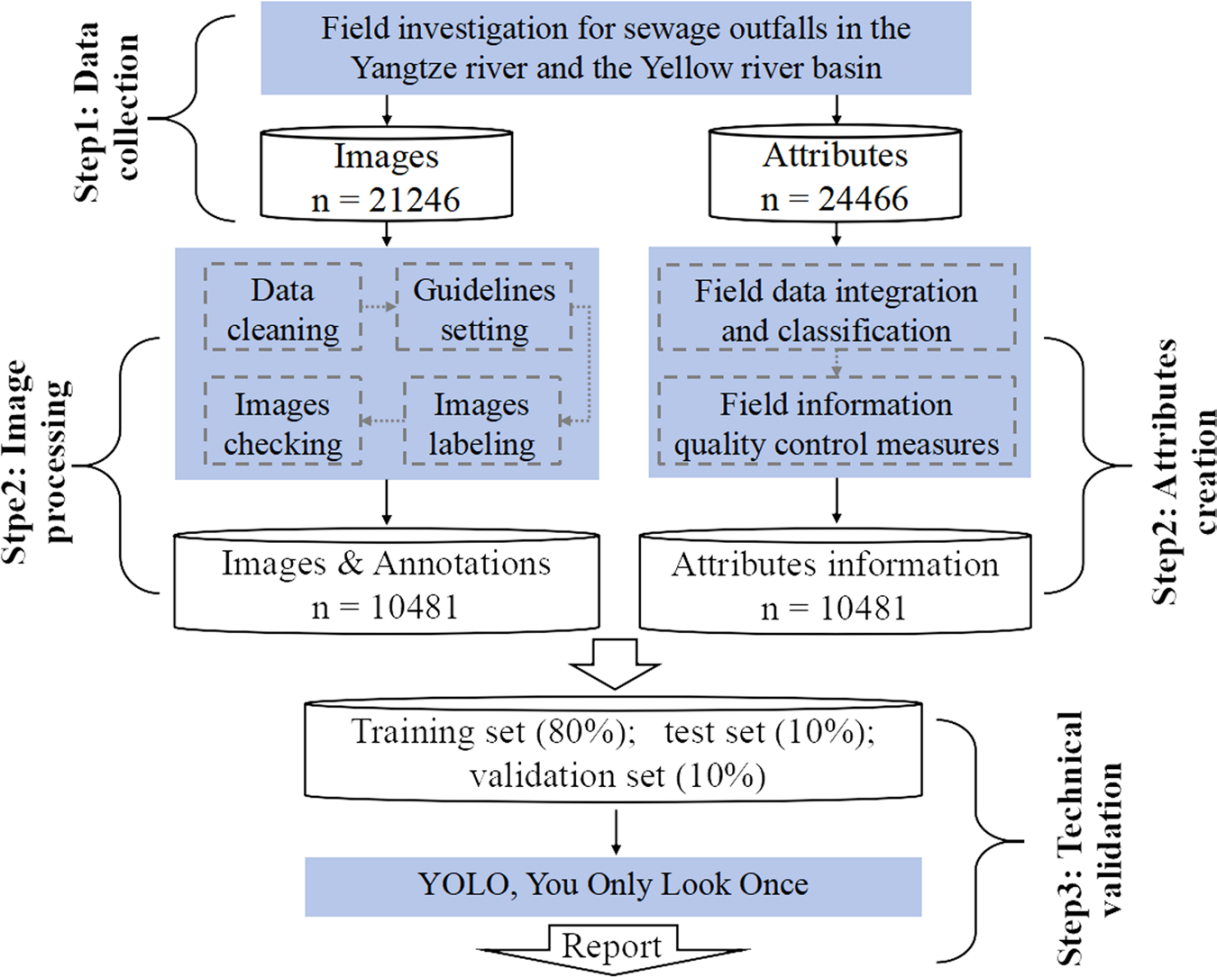
Schematic overall of the iSOOD datasets creation and technical validation.

Figure 1 illustrates the essential steps involved in the development of iSOOD datasets.

### Data collection

21246 images and 24466 attributes were acquired from original field investigations conducted in China in the Yangtze River basin and the Yellow River basin. Field investigations were conducted in the same regions using UAVs and handheld cameras. As a result, hidden and frequently unnoticed SOs were identified.

### Image processing and attribute creation

Following acquiring original data, this study eliminates redundant, low-resolution images. Furthermore, annotation guidelines for iSOOD datasets were established^16^. This study used the Autodistill tool package in the Roboflow platform for labelling work^17^. This technique automatically applies pre-annotations to the SOs. The preliminary annotation findings allow our researchers to efficiently prioritize the SOs in the photos and make necessary modifications to the unsatisfactory annotations. To guarantee the exceptional quality of iSOOD, this study implemented a multi-level quality inspection approach. Each image must undergo review by at least two independent groups of researchers. Furthermore, the potential labelling mistakes are addressed through regular reviews. When confronted with ambiguous or contentious annotations, the researchers would engage in thorough discussion and ultimately reach a consensus. Finally, the first generation of the iSOOD dataset has been released, consisting of 10481 images of high quality, together with corresponding attributes.

The factors leading to the differences and changes in the quantity of images and attributes are as follows. (i) The differences between 21246 in the initial image files and 24466 in the original attributes arise due to numerous SOs in certain images. It is worth noting that this study merged the attributes to achieve a one-to-one correspondence between the SOs and the attributes. This ensures that the iSOOD dataset has both a single SO and multiple SOs images. (ii) The changes between the original SOs and the final SOs arise because some images are mistaken for the SOs, such as drinking water pipes and blurred images.

### Technical validation

The iSOOD with 10481 images was split into a training set (80%), test set (10%), and validation set (10%). These sets were utilized to train the YOLOv10 series models, and the technical verification was reported based on the obtained performance.

## Data Records

The iSOOD is freely shared via the Zenodo platform^18^. The iSOOD dataset consists of an image dataset in YOLO format accompanied by annotation files and attribute information in Excel format. Each row represents a single record of a sewage outfall at a specific location. The columns in the dataset are as follows:

1. **Image_name**: Corresponds to the image file name in the dataset (sequentially numbered starting with 1, such as 1. jpg, 2. jpg).
2. **Outfall_code**: Every outfall has a unique code.
3. **basin**: River basin affiliation (1 = Yangtze River, 2 = Yellow River).
4. **typ**: Type of sewage outfalls. (1 = Combined sewer, 2 = Rainwater, 3 = Industrial wastewater, 4 = Agricultural drainage, 5 = Livestock breeding, 6 = Aquaculture, 7 = Surface runoff, 8 = Wastewater treatment plant, 9 = Domestic wastewater (e.g., wastewater not collected by wastewater treatment plants), 10 = Other).

### Statistics and examples of the dataset

**Fig. 2:**
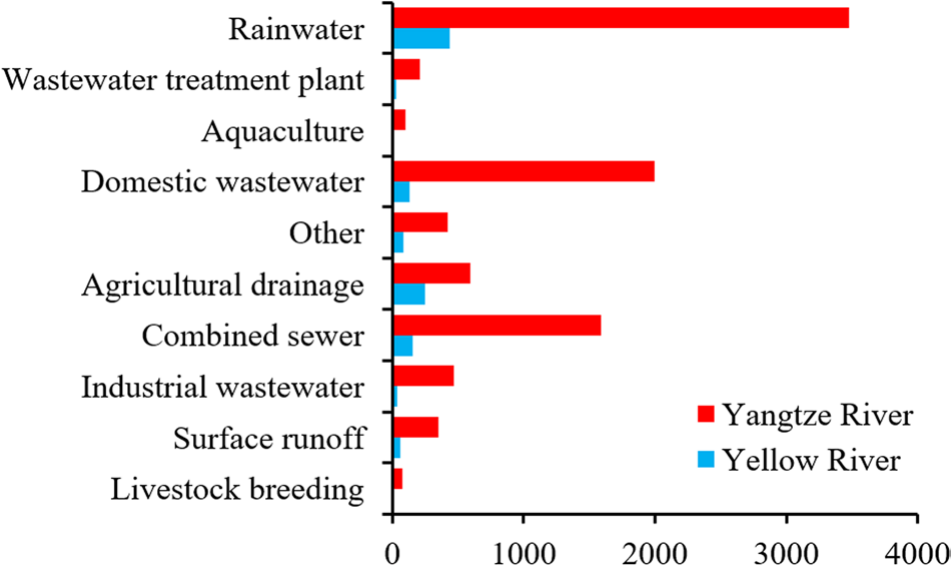
Categories of sewage outfalls in the iSOOD dataset.

**Fig. 3:**
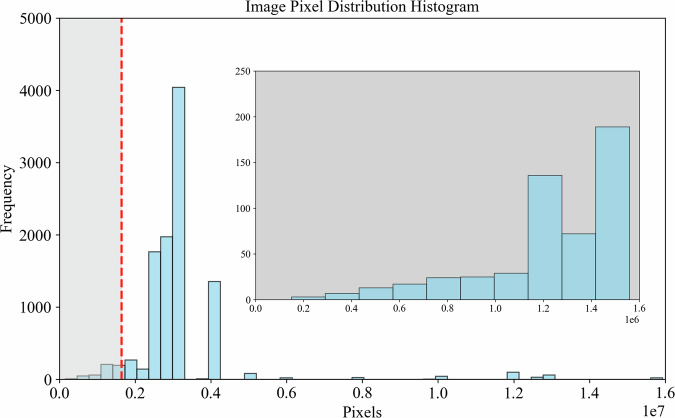
Pixel distribution histogram of images.

**Fig. 4:**
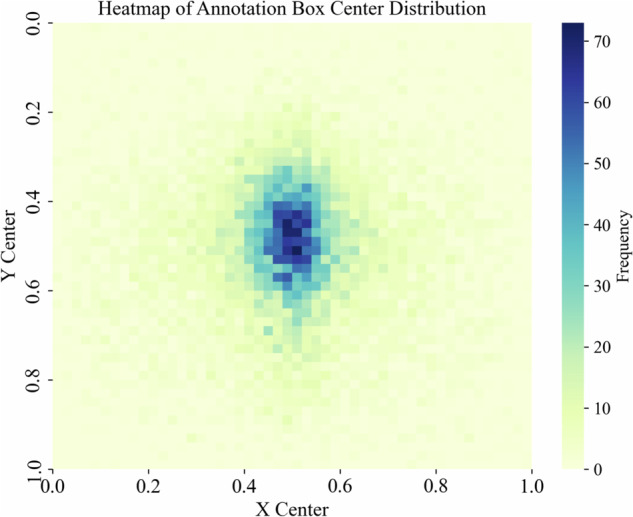
Heatmap of Annotation Box Center Distribution.

**Fig. 5:**
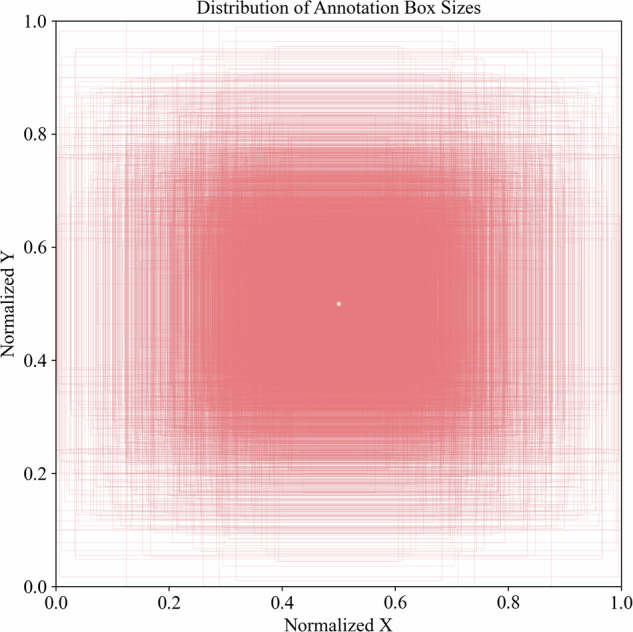
Heatmap of Annotation Box Distribution.

**Fig. 6:**
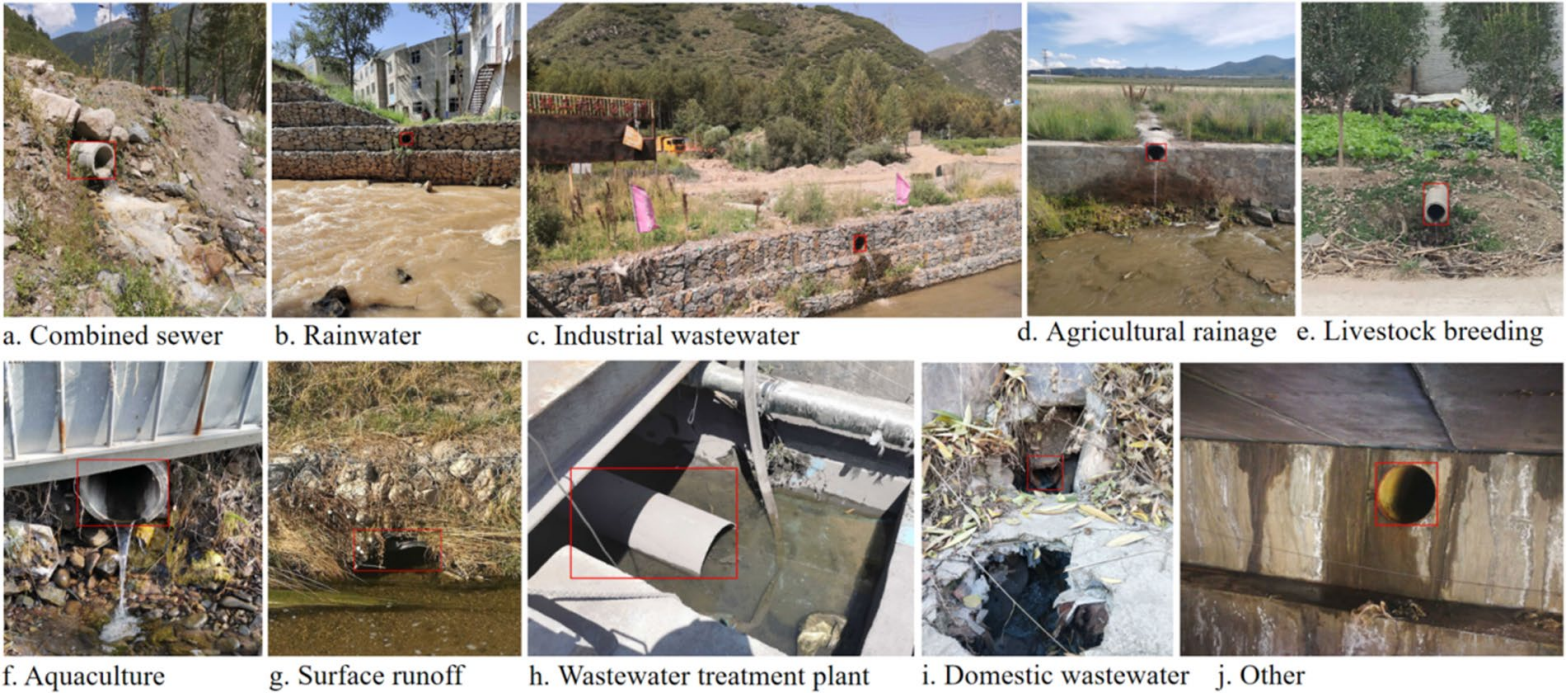
Example of sewage outfall classification.

**Fig. 7:**
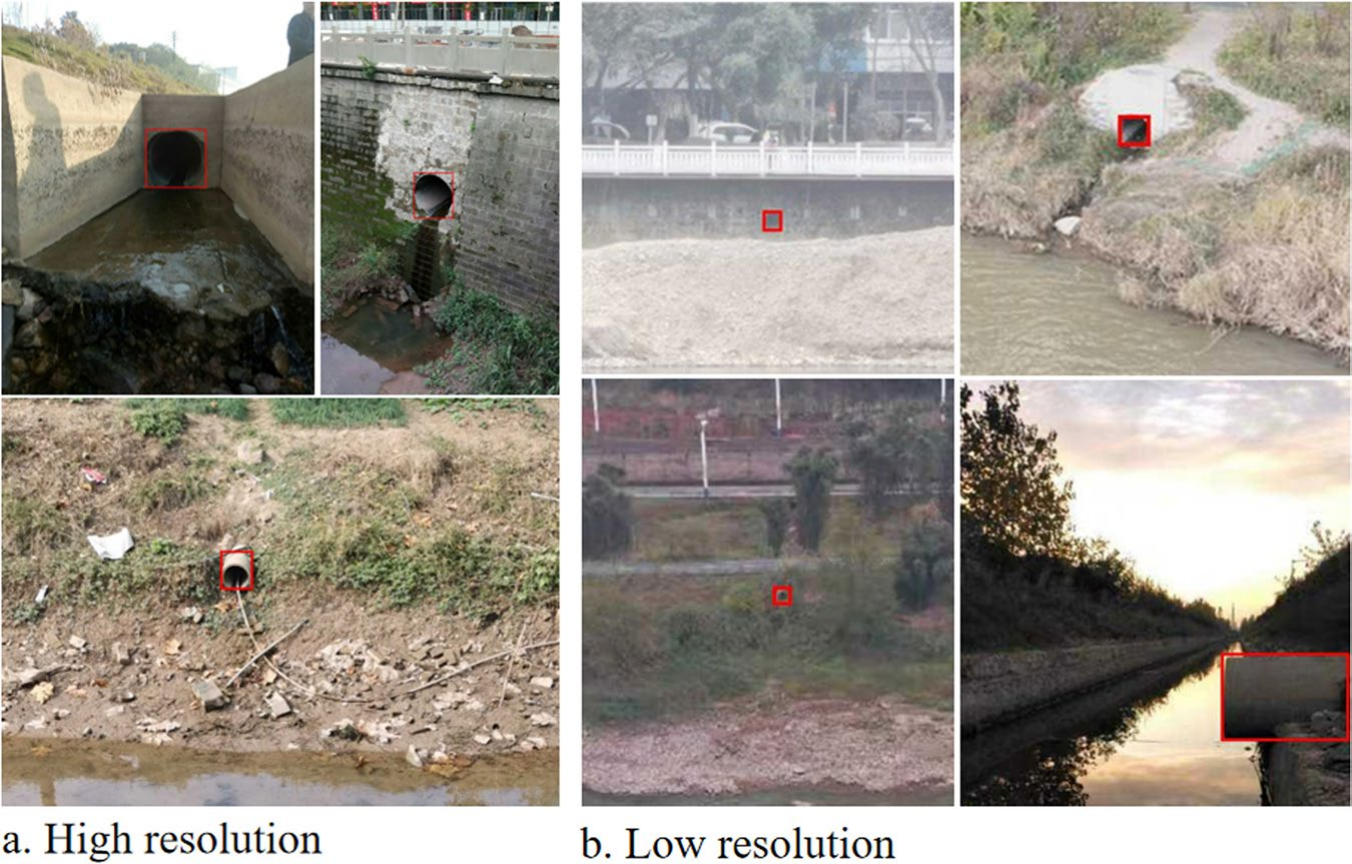
Example of sewage outfalls in different resolutions. High resolution refers to pixel values higher than 1280*1280; low resolution refers to pixel values lower than 1280*1280.

**Fig. 8:**
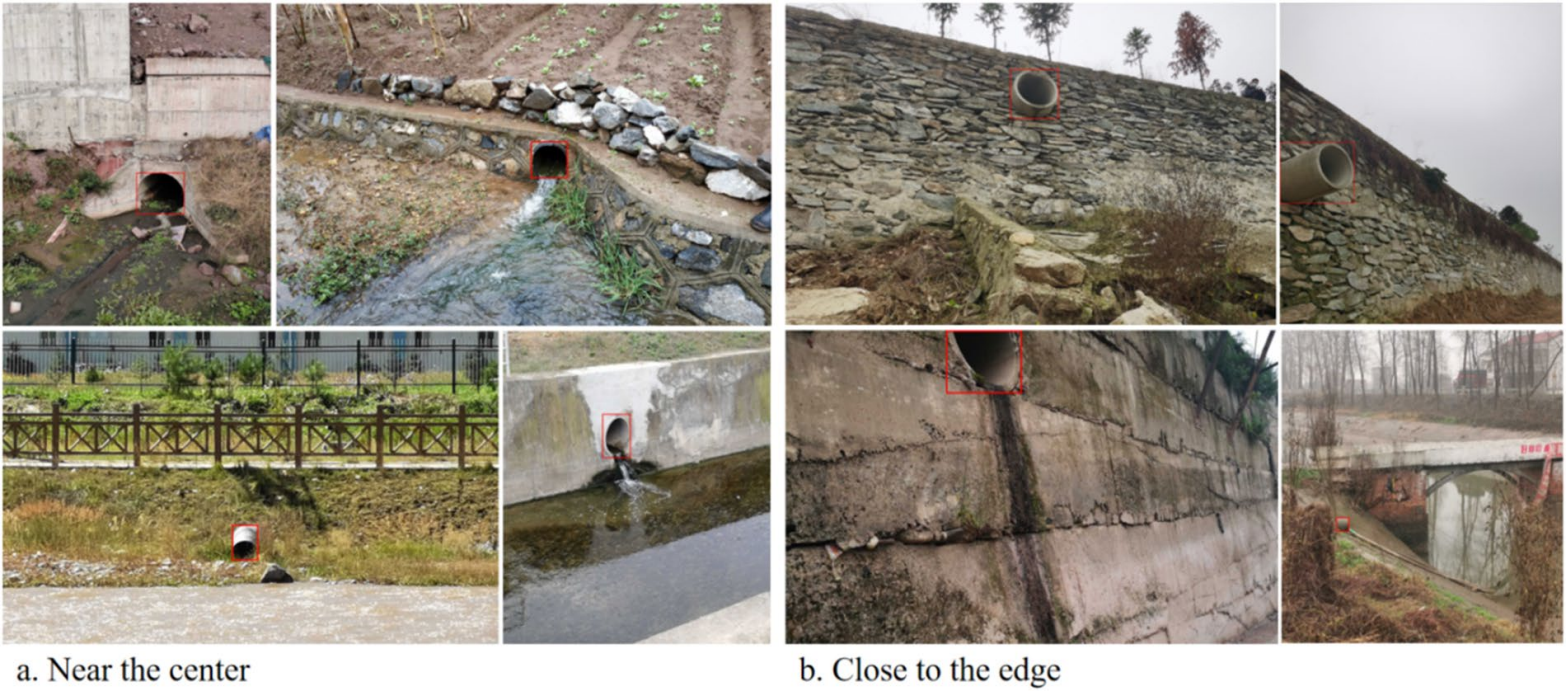
Examples of annotation distribution bounding boxed across sewage outfalls in the pictures. Near the centre means.25 $\le$ x_center $\le$ 0.75 and 0.25 $\le$ y_center $\le$ 0.75; Others means the close to the edge.

**Fig. 9:**
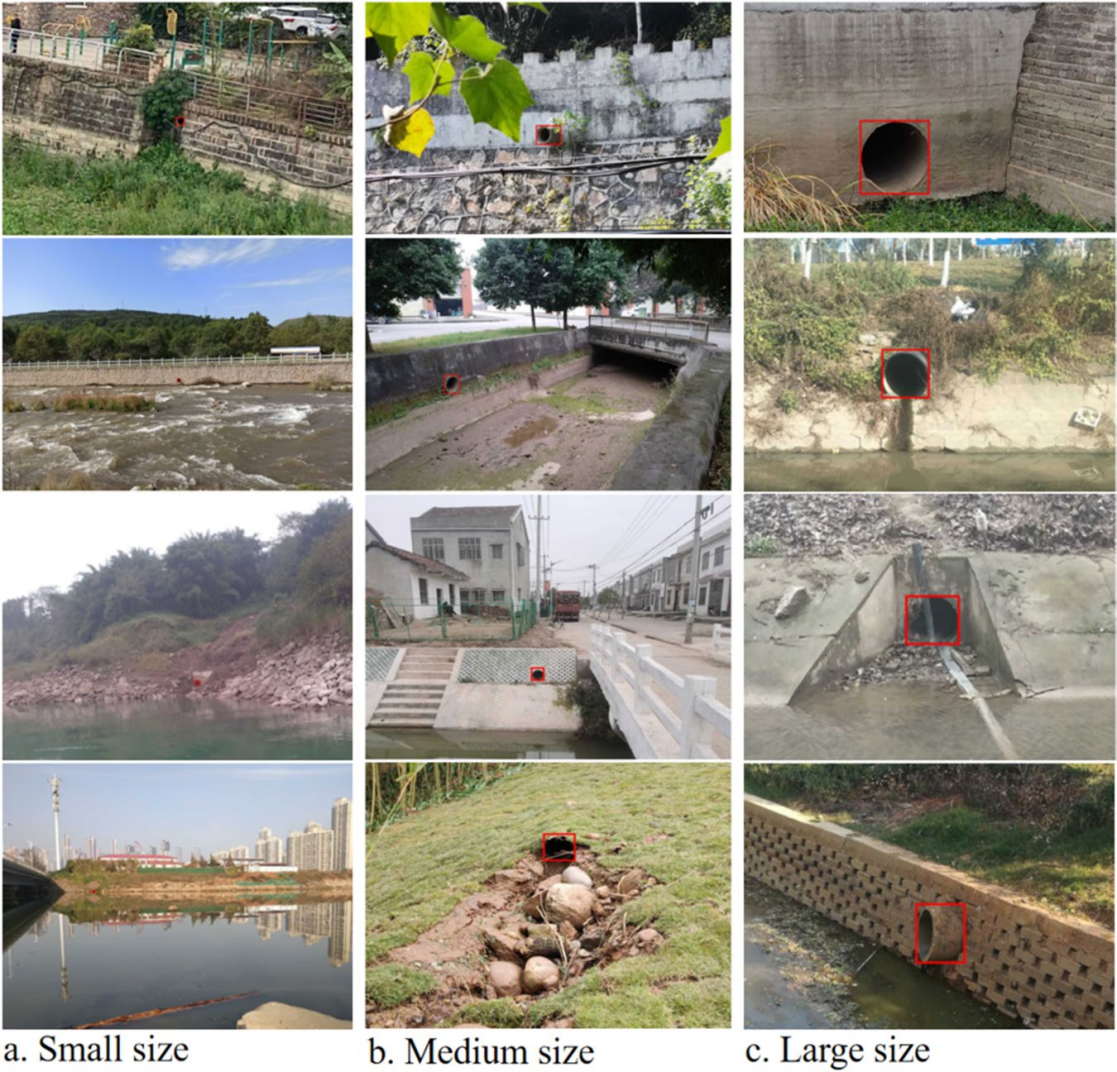
Example of sewage outfalls in different sizes. Small size refers to pixels below 32*32; medium size refers to the range from 32*32 to 96*96; and large size is above 96*96.

The iSOOD dataset has a total of 10481 SOs images in YOLO format, which were collected from the Yangtze River (9285 SOs images) and the Yellow River (1196 SOs images) in China (Fig. 2). The iSOOD dataset contains around ten types of SOs, as shown in Fig. 6. These SOs images in the iSOOD dataset have a range of pixel distribution (Fig. 3). Approximately 95.1% of the images in the iSOOD dataset have high pixels (Fig. 7). Figure 4 displays the heatmap illustrating the annotation box centre distribution of the iSOOD dataset. 77.3% of the images depict SOs located near the centre of the picture, while the remaining 22.7% show SOs around the edge area (Fig. 8). The number of large-sized SOs object images with pixel values greater than 96*96 is the largest, accounting for 80.0% of the iSOOD dataset. The second largest is the medium-sized SOs targets (accounting for 18.6%), and the smallest is the small-sized SOs targets with pixel values less than 32*32 (accounting for 1.4%) (Fig. 5). The original size of the annotation boxes and SOs is shown in Fig. 9.

## Technical Validation

### Environment settings

Given the potential application scenario of using the UAVs for real-time detection of SOs, the deep learning model must have the characteristics of rapid target recognition and compatibility with low-version hardware ports. Therefore, the YOLOv10 series, one of the state-of-the-art target detection models, was used to evaluate the performance of the iSOOD dataset. Compared with the previous YOLOv series, the YOLOv10 series has faster speed and higher accuracy^13^. The iSOOD dataset, comprising 10481 images of SOs, was randomly split into a training set (80%), test set (10%) and validation set (10%)^19^. The training of the YOLOv10 series utilized a personal computer with RTX 3090 24GB GPU, employing the default hyper-parameters. The batch sizes for the series were set to 16. The training epochs for the YOLOv10 series were set to 100. Before training, the iSOOD images were randomly translated, flipped, and scaled.

### Evaluation metrics

This study evaluates the efficacy of integrating the iSOOD dataset with the YOLOv10 series. We utilized the *pycocotools* to extract the average precision (AP) and the average recall (AR) metrics^20^. The AP metric evaluates a model’s capacity to accurately identify relevant objects by quantifying the proportion of actual positive detection. The AR metric evaluates the model’s ability to detect all relevant cases by measuring the percentage of actual positive detection among all relevant ground truths. The metrics evaluate the model’s ability by comparing the bounding boxes identified by the model with the annotation bounding boxes of the SOs^21^. The higher the values of AP and AR, the more satisfactory evaluation outcomes. The Intersection over the Union (IoU) threshold is a critical parameter that significantly impacts the evaluation results. The AP@50:5:95 is calculated at different IoU thresholds, typically from 0.5 to 0.95, with a step size of 0.05. The AP50 and AP75 are calculated with IoU values set to 0.50 and 0.75, respectively^22^. Furthermore, the study assessed the detection performance of images of various sizes of SOs^[]^. The AR01, AR10, and AR100 represent the average AR of 1, 10, and 100 maximum number detection objectives for all IoU thresholds.

### Performance evaluation

Table 1 shows the performance evaluation for the YOLOv10 series at different IoU thresholds. The training, validation and testing performances demonstrated that there was no over-fitting. On the test dataset, the AP and AR metrics are 0.626~0.883 and 0.597~0.785, which are better than the results of previous studies^10,12^. These results indicate that the iSOOD dataset is suitable for developing a deep learning model to utilise SOs objective detection in natural environments effectively. Table 2 shows the performance evaluation for the YOLOv10 series at different sizes of SOs objectives. On the test dataset, the AP and AR metrics for small-size SOs objectives range are 0.078~0.196 and 0.236~0.336, indicating a relatively low accuracy in identifying small-sized objectives. This is not related to the limitation of the models because the feature information contained in small-sized SOs images is very sparse. To ensure the precision of SOs detection in practical applications, one effective method is to operate the UAVs close to the river bank to take high-resolution images of SOs^23^.

**Table 1:** Performance evaluation for YOLOv10 series at different IoU thresholds.

Model	Dataset	AP			AR		
		AP@50:5:95	AP50	AP75	AR01	AR10	AR100
YOLOv10n	train	0.753	0.938	0.857	0.705	0.814	0.829
	valid	0.646	0.865	0.719	0.620	0.738	0.769
	**test**	**0.626**	**0.848**	**0.712**	**0.597**	**0.720**	**0.753**
YOLOv10s	train	0.821	0.970	0.917	0.759	0.859	0.867
	valid	0.671	0.887	0.756	0.647	0.750	0.780
	**test**	**0.659**	**0.871**	**0.736**	**0.626**	**0.741**	**0.772**
YOLOv10m	train	0.850	0.977	0.941	0.783	0.882	0.888
	valid	0.688	0.902	0.765	0.654	0.764	0.788
	**test**	**0.666**	**0.873**	**0.739**	**0.631**	**0.748**	**0.777**
YOLOv10b	train	0.862	0.979	0.948	0.793	0.892	0.896
	valid	0.689	0.898	0.768	0.654	0.766	0.790
	**test**	**0.667**	**0.871**	**0.740**	**0.633**	**0.749**	**0.775**
YOLOv10l	train	0.874	0.981	0.953	0.802	0.899	0.903
	valid	0.703	0.906	0.779	0.668	0.771	0.796
	**test**	**0.679**	**0.883**	**0.750**	**0.640**	**0.762**	**0.783**
YOLOv10x	train	0.882	0.984	0.959	0.809	0.907	0.910
	valid	0.710	0.910	0.79	0.664	0.782	0.806
	**test**	**0.687**	**0.883**	**0.759**	**0.65**	**0.754**	**0.785**

**Table 2:** Performance evaluation for YOLOv10 series at different sizes of sewage outfalls objectives.

Model	Dataset	AP			AR		
		Small	Medium	Large	Small	Medium	Large
YOLOv10n	train	0.221	0.603	0.794	0.366	0.712	0.864
	valid	0.157	0.541	0.680	0.300	0.692	0.797
	**test**	**0.078**	**0.511**	**0.668**	**0.236**	**0.654**	**0.789**
YOLOv10s	train	0.281	0.673	0.860	0.422	0.755	0.900
	valid	0.192	0.603	0.698	0.328	0.727	0.801
	**test**	**0.101**	**0.566**	**0.696**	**0.241**	**0.690**	**0.804**
YOLOv10m	train	0.283	0.714	0.886	0.416	0.785	0.919
	valid	0.244	0.602	0.718	0.372	0.730	0.811
	**test**	**0.121**	**0.567**	**0.705**	**0.273**	**0.679**	**0.813**
YOLOv10b	train	0.304	0.731	0.897	0.441	0.792	0.928
	valid	0.243	0.611	0.719	0.356	0.725	0.815
	**test**	**0.108**	**0.581**	**0.704**	**0.286**	**0.680**	**0.811**
YOLOv10l	train	0.301	0.740	0.909	0.430	0.798	0.935
	valid	0.256	0.623	0.731	0.361	0.737	0.819
	**test**	**0.196**	**0.606**	**0.712**	**0.336**	**0.698**	**0.814**
YOLOv10x	train	0.335	0.757	0.916	0.449	0.811	0.940
	valid	0.202	0.646	0.737	0.372	0.742	0.830
	**test**	**0.128**	**0.589**	**0.727**	**0.277**	**0.693**	**0.819**

## Usage Notes

This study presents the first fine-grained dataset for SOs objective detection in natural environments. The iSOOD includes 10481 images captured by UAVs and handheld cameras. Our researchers meticulously annotated the iSOOD to assign labels to SOs. The iSOOD have been publicly released after desensitization to promote interdisciplinary collaboration and accelerate advancements in intelligence watershed management. We expect the iSOOD dataset to inspire further research on the SOs detection and the control of pollution migration paths and serve as a fundamental resource for using advanced deep learning visual technology in environmental monitoring.

### Implications of the iSOOD for intelligence watershed management

The importance of SOs inspection in improving the water ecological environment has gradually attracted the recognition of policymakers. There is a significant demand for iSOOD and related technology in watershed management. For example, the Chinese administration is initiating an in-depth investigation into SOs across the country. China has allocated billions of dollars and employed tens of thousands of knowledgeable employees only in the Yangtze River and Yellow River basins’ upper and middle sections. Nevertheless, the ongoing investigation of the SOs constitutes only about 10% of the overall effort. Internationally, countries can also examine the “China model” to investigate the SOs within the river basin to guarantee the water’s ecological safety. The extensive application scenarios mean that iSOOD and related intelligence technologies have great potential to replace manual labour in SOs detection, significantly reducing costs and enhancing efficiency.

The most important recommendation is to implement artificial intelligence technologies related to iSOOD on the UAV platform for watershed management. More precisely, the specific details are as follows. (i) There is a requirement for UAVs that can operate at low altitudes and be easily navigated, along with flight control algorithms that are compatible with these platforms. This study found that the precision of identifying minor SOs is relatively low. To cope with this challenge, a reliable UAV platform is required to acquire high-resolution images of SOs within its range of vision using automated cruse and near-up flights. (ii) The YOLO series algorithm architecture is the primary focus of application in artificial intelligence for automatically identifying SOs. Object detection techniques can be classified into two-stage and one-stage algorithm methods. As a one-stage algorithm, the YOLO series offers the benefit of rapid processing, making it particularly well-suited for real-time surveillance^24,25^. Nevertheless, compared to the standard two-stage approach, YOLO also has the drawback of reduced detection accuracy^26–28^. Hence, it is imperative to conduct further research to enhance the detection speed and accuracy of algorithms built upon YOLO. (iii) The iSOOD dataset could be able to continuously gather and accumulate images to enhance its performance in tasks associated with SOs detection worldwide.

## Data Availability

The Python 3.9 scripts used for generating statistics presented in the article, as well as the code for validating the completeness of the dataset (including YOLOv5 and YOLOv10), are available at https://github.com/Daniel00ll/iSOOD-code.
